# Update on neurological symptoms in patients infected with severe acute respiratory syndrome coronavirus‐2

**DOI:** 10.1002/ibra.12008

**Published:** 2021-12-20

**Authors:** Mei‐Fang Xiao, Zhi‐Jian You, Chang Zeng, Ze‐Bing Huang, Liang Dong

**Affiliations:** ^1^ Health Management Center, Xiangya Hospital Central South University Changsha Hunan China; ^2^ National Clinical Research Center for Geriatric Disorders, Xiangya Hospital Central South University Changsha China; ^3^ Department of Anesthesiology Liuzhou People's Hospital Affiliated to Guangxi Medical University Liuzhou Guangxi China; ^4^ Department of Infectious Diseases, Xiangya Hospital Central South University Changsha Hunan China; ^5^ Key Laboratory of Viral Hepatitis Changsha Hunan China

**Keywords:** ACE‐2 receptors, COVID‐19, neurological symptoms, pathological mechanisms

## Abstract

Novel coronavirus 19 (COVID‐19) is the latest and most intense epidemic, which is caused by the severe acute respiratory syndrome coronavirus‐2 (SARS‐CoV‐2). In addition to causing respiratory symptoms, SARS‐CoV‐2 can have severe effects on the nervous system. Clinically, COVID‐19 patients have been reported ranging from mild hypogeusia and hyposmia to severe neurological disorders, such as encephalopathy, encephalitis, strokes, and seizures syndrome. However, the pathological mechanisms of this SARS‐CoV‐2 neuro aggressiveness remain unclear, so it is of great significance to explore the neurological effects of SARS‐CoV‐2 infection. To facilitate clinicians to timely recognize the manifestations of COVID‐19 patients with neurological injury and timely treatment, the author hereby reviews the latest research progress in the possible pathways, clinical manifestations, and pathogenesis of COVID‐19 patients with nerve injury.

## Graphical abstract

Severe acute respiratory syndrome coronavirus‐2 can cause nervous system injury in the human body by direct, hypoxia, and immune injury, which affects a part of novel coronavirus 19 patients with strokes, seizures, myelitis, as well as other neurological complications.

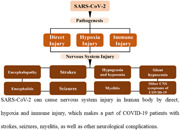

## 1 INTRODUCTION

In December 2019, the novel coronavirus 19 (COVID‐19) outbreak appeared, and then COVID‐19 spread in local areas and quickly spread around the world, causing global concern.^1^ In February 2020, COVID‐19 was caused by SARS‐CoV‐2, officially named by the World Health Organization. Previous studies have found that the most common symptoms for COVID‐19 patients were upper respiratory symptoms (such as cough, fatigue, fever, and often pulmonary infection).^2^ While the most prominent manifestations of patients with COVID‐19 are respiratory diseases, reports of neurological manifestations have increased markedly. The latest study found that in addition to respiratory symptoms, approximately 36% of COVID‐19 patients also developed neurological symptoms, leading to the development of the corresponding neurological disease. These clinical presentations may be a combination of nonspecific central nervous system (CNS) symptoms, the effects of viral infection, or vasculature and nervous system inflammation with side‐ or postinfectious mechanisms.^3^ It can be speculated that the pathology of the observed clinical symptom may be due to a novel immune response or direct infection. Based on what is known about other coronaviruses, it is not surprising that COVID‐19 has extensive CNS complications because many reports have suggested these associations.^4^ Through well‐designed diagnostic, clinical and epidemiological studies, CNS symptoms with COVID‐19 can be better confirmed and observed to determine whether the symptoms are causative or coincidental.^5^ Therefore, with the continuous global COVID‐19 pandemic, the number of CNS diseases caused by SARS‐CoV‐2 infection have increased significantly, so it has been paid more and more attention by the first‐line clinicians.^6^

Many studies found that once the virus enters the body, it may invade CNS through hematogenous dissemination.^7^ Viruses may also enter the CNS via retroactive neurotransmission, where viruses infect peripheral nerve cells and spread to the spine and brain through existing neurotransmission mechanisms. This review's purpose is to summarize the reported neurological symptoms of COVID‐19 and to further review the pathological mechanisms that cause these symptoms.

## 2 COVID‐19 CAUSES NEUROLOGICAL DISEASE

Neurological symptoms were sporadic in patients with COVID‐19 in current studies, but have not been fully studied.^8^ Numerous studies have shown that the SARS‐CoV‐2 virus can influence a patient's nervous system in a variety of different ways.^9^ This virus undoubtedly possessed the neuroinvasive ability but remained to be further studied. A recent study has found that the most common neurological symptoms, such as headache, dizziness, and fatigue, for COVID‐19 patients were nonspecific symptoms.^10^ In a study of 417 COVID‐19 patients, there are 86% of the patients with olfactory dysfunction and 88% with taste problems after treatment. Actually, anosmia is the first symptom of COVID‐19 in 12% of patients.^11^ Anosmia is more common in COVID‐19 patients than patients with other viruses.^12^

Meanwhile, researchers found that the occurrence of neurological clinical symptoms appeared to be closely related to the severity of COVID‐19. In a study of more than 200 hospitalized COVID‐19 patients, there were significantly more reports of neuro‐related symptoms in severely ill patients than in nonseverely ill patients. In this study, the most common neurological symptoms were an acute cerebrovascular disease, alienation of self‐consciousness, and muscle injury.^13^

Most of the COVID‐19 patients seem to have pulmonary symptoms before the appearance of their neurological symptoms. About 30% of patients with COVID‐19 faced a variety of neurological complications, which included headache, dizziness, impaired consciousness, and so on. In addition to impaired smell and taste, visual impairment has also been widely reported. Health care workers detected SARS‐CoV‐2 nucleic acid in the patient's cerebrospinal fluid (CSF), and this virus was also found in brain tissue during autopsies of patients who died of COVID‐19.^14^ Although these symptoms are relatively rare, SARS‐CoV‐2 virus has been shown to significantly affect the nervous system of the body. The researchers also found SARS‐CoV‐2 in the CSF of COVID‐19 female patients through polymerase chain reaction testing.^15^

### 2.1 Encephalopathy

Encephalopathy, usually manifested as delirium, is an acute or subacute disorder of the brain that results in an altered state of consciousness or mind.^16^ Therein, older adults with high blood pressure are at higher risk for changes in their mental status when they are infected by COVID‐19. Researchers have now found brain edema in COVID‐19 victims, a dangerous condition that can lead to increased intracranial pressure and encephalopathy.^17^ COVID‐19 patients with a disease of nerve damage are at higher risk for encephalopathy as the initial symptoms.^18^ Compared with the risk of COVID‐19 progression, there is a strong relationship between the risk of delirium and age. When people with COVID‐19 are isolated from their families and are alone, they may feel desperate and panicked.^19^

Acute necrotizing encephalopathy (ANE) and hemorrhagic lesions were also observed in COVID‐19 patients.^20^ Another 50‐year‐old COVID‐19 patient, who suffered from aplastic anemia, primarily presented with brain stem involvement.^21^ Although ANE occurs in an infected environment, researchers do not believe it is caused by a neuroinvasive disease. Currently, the exact pathophysiology of ANE remains unclear, it can be speculated that it may be caused by genetically susceptible individuals with cytokine‐mediated brain damage.

A male patient with COVID‐19 presented with a rather depressed mental state and occasional diffuse myoclonic movements following hypoxia. Examination of the case showed that his pupils were sluggish and there was no response to harmful stimuli in the extremities. The computed tomography (CT) of his head showed a symmetrical fusion of low‐density shadows containing supratentorial white matter. His magnetic resonance imaging (MRI) demonstrates extensive white matter demyelination with active demyelination and putative necrosis of the central region.^22^

The researchers speculate that delirium in hospitalized patients with COVID‐19 may be due to an attack on CNS, induced by inflammatory mediators of the CNS, secondary to failure of other organ systems and sedative effects, which may be due to psychological manifestations or environmental factors.^23^ In COVID‐19 patients, delirium due to direct entry of the SARS‐CoV‐2 virus into the CNS is possible and may be accompanied by certain symptoms, such as seizures, increased intracranial pressure, and so forth.^24^ To date, delirium in patients with COVID‐19 has been under‐reported, and in fact, delirium in various conditions is considered to be generally under‐reported unless it is expressly supervised.^25^ Overall, delirium occurred in 70% of COVID‐19 patients admitted to intensive care unit (ICU), suggesting that delirium may be closely related to long‐term cognitive dysfunction.

Toxic encephalopathy, that is, acute toxic encephalitis, is a brain dysfunction syndrome caused by systemic toxemia, metabolic disorders, and hypoxia. The clinical symptoms of toxic encephalopathy are very varied, including restlessness and mental disorders in mild cases, and even dizziness and paralysis in severe cases.^26^ Acute viral infections, such as respiratory infections caused by a coronavirus, are an important cause of the disease.^27^ Based on this, we can infer that COVID‐19 patients often experience severe hypoxia, which leads to toxic encephalopathy. In addition, nearly 35% of COVID‐19 patients presented the symptoms of headache, consciousness disturbance, and other brain dysfunction.^28^ The autopsy reports from some COVID‐19 patients showed edema in the brain tissue of these patients.^29^ The above studies can provide sufficient evidence for toxic encephalopathy caused by COVID‐19.

ANE is a very rare complication of other viral infections. COVID‐19 virus infection can lead to a destruction of the blood–brain barrier (BBB), and then leads to nervous system diseases.^30^ Although the disease mainly occurs in children, it also occurs in adults and is characterized by symmetrical multifocal lesions with constant thalamic damage. Although ANE mainly occurs in children, it also occurs in adults and is characterized by symmetrical multifocal lesions with constant thalamic damage. The first reported COVID‐19‐related ANE was a patient who tested positive for the virus, whose cranial magnetic resonance showed an enhanced signal of the bleeding margins in the bilateral thalamus, and middle temporal lobe, consistent with the findings of ANE.^31^ Dixon also reported a patient of ANE aplastic anemia associated with COVID‐19, whose imaging findings were mainly brain stem involvement.^21^

### 2.2 Encephalitis

Small amounts of meningitis and encephalitis have been reported in COVID‐19 patients, but at this stage, it is still unclear which immune‐mediated diseases these are. Encephalitis is considered to be an inflammatory lesion of brain parenchyma caused by pathogens, which mainly damage the patients' neurons and nerve tissue. Currently, clinical data about COVID‐19 show that many COVID‐19 patients present headaches, disturbance of consciousness, and other symptoms similar to intracranial infection.^32^ Karvigh reported on a patient with COVID 19‐related encephalitis who developed fever convulsions and disturbance of consciousness, even having multiple seizures during this period. The corresponding imaging results displayed high signal changes hippocampus and lateral ventricle. The researcher found positive for SARS‐CoV‐2 virus through CSF tests, further confirming the possibility that COVID‐19 infection may lead to meningitis.^33^ A Chinese male COVID‐19 patient had encephalitis, but medical personnel found no trace of SARS‐CoV‐2 in his CSF. This patient's symptoms changed consciousness, and his encephalitis had gradually recovered after COVID‐19 treatment.^34^ Although a part of COVID‐19 patients may develop into encephalitis, this phenomenon is not common at this stage.^35^

In addition to the typical encephalitis described above, some specific types of encephalitis have also been reported. For example, Efe et al. found a rare patient of encephalitis related to COVID‐19, which was shown to be high‐grade glioma by radiological examination, while it was indicated to be encephalitis by histological examination, and the SARS‐CoV‐2 test of this case was positive.^36^ Recently, Domingues et al. further confirmed the presence of SARS‐CoV‐2 in CSF of COVID‐19 patients through genome sequencing, which clinically confirmed the possibility of COVID‐19 virus causing encephalitis.^37^ Numerous research have shown that COVID‐19 patients experience ataxia and convulsions in which some patients experience changes in their mental status, some patients experience neck stiffness, and some patients experience meningeal irritation.^38^, ^39^

Encephalitis in patients with COVID‐19 may also present as seizures. Of particular note is the possibility that such patients may exhibit a nonconvulsive epileptic status. The Salzburg Consensus Standards for Non‐Convulsive Epileptic Status, which was published in 2015, provide a definition of the terms associated with NCSE and identify specific EEG tracings.^40^ Currently, researchers found that between 0.7% and 1% of COVID‐19 patients in coma occurred the complications of epilepsy.^41^ Thus, the above studies can provide a certain clinical basis for the COVID‐19 virus to cause encephalitis.

### 2.3 Strokes

The studies found a significant interaction between stroke and COVID‐19. The severe COVID‐19 patients increase in multiple vascular risk factors as the length of hospital stay increases. Stroke has gradually become an increasingly important adverse event in patients with COVID‐19. Chinese research reported that acute cerebrovascular disease accounted for 2.8% of approximately 200 COVID‐19 patients, including four strokes and one cerebral hemorrhage.^42^ Meanwhile, the researchers also found an approximately 5.1% incidence of cerebrovascular disease in 138 patients with COVID‐19.^43^ A recent study reported that five patients with confirmed COVID‐19 infection presented with cerebral stroke symptoms.^44^

Strokes usually manifest as ischemic and hemorrhagic strokes.^45^ Goldberg reported the first patient with COVID‐19‐related cerebral infarction, whose clinical manifestations were consistent with COVID‐19‐induced acute respiratory distress syndrome (ARDS), indicating acute cerebral infarction; meanwhile, the laboratory indicators suggested elevated white blood cell count and hypercoagulability.^46^ A research of about 184 COVID‐19 patients showed that three patients experienced ischemic stroke.^47^ In recent research, three COVID‐19 patients presented with multiple arterial thromboses in the upper and lower extremities of the brain.^48^

In a research of 33 patients who died from COVID‐19 intracerebral hemorrhage, 15.2% of those who died had large hematomas with diffuse hypoxic‐ischemic injury.^49^ A study by Kremer et al. showed that 11 (30%) COVID‐19 patients had hemorrhagic lesions and nine (24%) isolated white matter microbleeding, respectively.^50^ A COVID‐19 old man showed massive intracerebral hemorrhage and subarachnoid hemorrhage through a head CT scan.^51^ Therefore, COVID‐19 patients should always be vigilant for acute cerebrovascular events.

### 2.4 Seizures

Due to electrolyte disturbances, organ failure, and brain damage in COVID‐19 patients, these patients are at risk for seizures. A COVID‐19 patient developed generalized tonic‐clonic symptoms; however, his MRI and CSF examinations were normal.^52^ The incidence and risk of acute symptomatic epilepsy were identified in a study of about 304 severe COVID‐19 patients. Although no seizures were observed in these COVID‐19 cases, clinical seizures in these cases may be missed because of the lack of real‐time EEG monitoring.^53^

In the COVID‐19 pandemic, staff exposure was reduced due to the reduced use of EEG; it is important to be aware that undetected nonconvulsive seizures may occur when patients are sedated. However, there is now a growing awareness that it is possible to try using rapid‐response EEG reduces additional contact with staff who are in the same room with the patients.^54^

EEG can be used for the clinical management of COVID‐19, especially in ICUs. In 22 COVID‐19 patients by a recent study, who were monitored by EEG for 24 h continuously, nearly 10% of them developed seizures.^55^ Hepburn reported two COVID‐19 patients with epileptic symptoms. EEG results revealed a rhythmic discharge in the right frontal center of the first patient with the corresponding clonic left arm movement. The second patient presented with a rhythmic discharge in the left frontotemporal region corresponding to the right facial clonic movement that spread back and forth.^56^ These studies further demonstrate the importance of EEG monitoring for COVID‐19 patients with seizures.

Currently, in the absence of sufficient evidence, an attempt should be made to diagnose, classify, and treat seizure complications in patients with COVID‐19.^57^ Therefore, when treating seizures in COVID‐19‐infected patients, medical staff in particular need to examine the interaction between antiepileptic and COVID‐19‐treated drugs.

### 2.5 Hypogeusia and hyposmia

Hypogeusia and olfaction are typical symptoms and major neurologic manifestations of COVID‐19.^58^ A large number of otolaryngologists have reported a large number of COVID‐19 patients with hyposmia, but current scientific data confirm that the relationship is limited.^59^ Anosmia may be due to SARS‐CoV‐2 transport to the brain near the olfactory bulb. MRI studies showed abnormal signals in the olfactory bulb and right posterior straight gyrus.^60^ About one third of hospitalized COVID‐19 patients showed some degree of impaired smell or taste.^61^ In another study of 417 European COVID‐19 patients, about 85% of them showed symptoms of impaired smell and taste.^62^

Research of 237 COVID‐19 anosmia patients shows that 73% of these patients found anosmia before COVID‐19 was diagnosed, while 26.6% initially had anosmia. About 27% of COVID‐19 patients showed some improvement in their anosmia within 7 days.^63^ Most (65.7%) of the patients reported olfactory dysfunction after the onset of general ear, nose, and throat symptoms; however, in another study, 11.8% of patients reported hyposmia before any other symptoms, indicating that anosmia may be important for early detection of COVID‐19.^64^

Most COVID‐19 patients have a sense of olfaction and gustation dysfunction that does not require specific treatment, and these symptoms will recover as COVID‐19 symptoms gradually improve. Approximately 80% of all COVID‐19 patients with smell and taste impairment are reported to regain their sense of smell and taste impairment within 2 weeks of the COVID‐19 cure.^65^

### 2.6 Myelitis

To our knowledge, very few cases of COVID‐19 with myelitis have been reported at this stage. Several recent reviews discussing neurologic complications of COVID‐19 patients have been reported, but myelitis is rarely mentioned,^66^ possibly due to the relatively low incidence of this complication in COVID‐19 patients. However, the incubation in COVID‐19 patients from the onset of myelitis may lead to misdiagnosis of patients.

Nonspecific characteristics of myelitis associated with COVID‐19 patients must use a wide range of differential diagnoses to solve diagnosis problems. When anosmia and bilateral pneumonia with stromal lesions occur in COVID‐19 patients, the cause of these symptoms must be suspected. However, other causes may produce similar symptoms, such as *Mycoplasma pneumoniae* myelitis, which can occur before different forms of respiratory infection.^67^ Researchers consider that myelitis is an infrequent but severe complication in patients with COVID‐19; meanwhile, complications of myelitis can cause patients with COVID‐19 to go from asymptomatic to the extreme, making a definitive diagnosis very challenging, which can make it more challenging to treat these patients.^67^

### 2.7 Silent hypoxemia

Researchers found that a part of COVID‐19 patients with hypoxemia did not experience breathing difficulties. Some COVID‐19 patients did not show dyspnea symptoms even when their oxygen saturation is below 80%.^68^ Relatively intact lung volume in the initial stages of the disease may explain the absence of breathing difficulties. CNS does not effectively increase voluntary and reflex respiratory drive due to the preservation of pulmonary compliance. For example, in older patients with COVID‐19, because they may have conditions such as diabetes and pulmonary thrombosis, this can lead to reduced breathing difficulties in these patients, followed by asymptomatic hypoxemia.^69^ Meanwhile, in some cases, oxygen saturation remains constant despite a lower arterial oxygen partial pressure, possibly due to the left shift in the oxygen–hemoglobin dissociation curve by hyperventilation.^70^

Currently, some researchers found that the reduced perception of COVID‐19 dyspnea may be explained by the following two mechanisms. First, neuromuscular dysfunction may be an important cause mechanism of respiratory insufficiency in COVID‐19 patients. In addition, the effect of cytokine storm and SARS‐COV‐2 on cortical margin through direct nerve invasion may be the other important mechanism that led to respiratory failure and asymptomatic hypoxemia.^71^ Based on the above analysis, silent hypoxemia was considered to be closely related to nervous system injury.

### 2.8 Other neurological symptoms of COVID‐19

One patient with schizophrenia was reported to present antipsychotic malignant syndrome after diagnosis of COVID‐19.^72^ Meanwhile, a recent study reported that an elderly female patient with COVID‐19 presented the complications of sensorineural hearing loss.^73^ In addition, a small number of COVID‐19 patients also showed other CNS symptoms that included Guillain‐Barré syndrome and Parkinson's disease.^74^ Although these CNS symptoms are rarely reported in patients with COVID‐19, these symptoms also need to be brought to the attention of relevant medical staff.

## 3 PATHOLOGICAL MECHANISMS OF NERVE DAMAGE IN PATIENTS WITH COVID‐19

The distribution of host receptor cells determines the directivity of the virus. In general, since these coronaviruses that are SARS‐CoV‐2 and SARS‐CoV, have similar microstructures and genetic structure, neurotropism may possess a similar characteristic of coronaviruses.^9^ Generally, receptor recognition is one of the most important steps in viral infection and pathogenesis. Some researchers found that the viral spike (S) protein of SARS‐CoV‐2 binds to the cell receptor of angiotensin‐converting enzyme‐2 (ACE2) in a similar manner to SARS‐CoV, but with a binding affinity 10–20 times greater. These results further suggest that increased ACE2 expression may facilitate the spread of SARS‐CoV‐2 in host cells and increase sensitivity to SARS‐COV‐2.^75^ Studies targeting angiotensin II receptor blockers (ARBs) and ACE inhibitors (AC‐I) have shown that upregulation of ACE2 expression in cells promotes SARS‐CoV‐2 binding and is associated with severe disease presentation. This receptor may be recognized by viral cells and host cells bind to the S protein induced by the host cell protease transmembrane protease serine 2 (TMPRSS2) into the virus. Angiotensin‐II clearance is significantly reduced by downregulation of ACE2 expression by SARS‐CoV‐2, resulting in increased tissue damage. In general, ACE‐2 receptors are highly expressed in the epithelial structure of human organs.^76^ Therein, TMPRSS2 activates spike (S) glycoprotein on the COVID‐19 membrane, and allows virions to bind ACE‐2. Activation of S glycoprotein is dependent on cathepsin B and L in endosomal cysteine proteases.^77^

**Figure 1:**
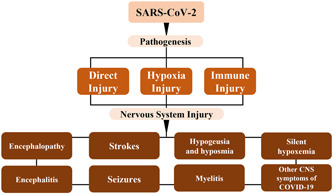
Schematic diagram of the pathogenesis of different neurological complications caused by SARS‐CoV‐2. CNS, central nervous system; SARS‐CoV‐2, severe acute respiratory syndrome coronavirus‐2 [Color figure can be viewed at wileyonlinelibrary.com]

Subsequently, the SARS‐CoV‐2 virus can influence the CNS through a variety of pathological mechanisms. Numerous research have indicated that ACE‐2 is not only found in astrocytes, oligodendrocytes and neurons, but also found in substantia nigra, ventricle, and olfactory bulb.^78^ The above reviews indicate that ACE‐2 is widely present in the human body, which leads the researchers to speculate that COVID‐19 may infect neurons in the CNS.^79^ The research by Dahm found that viruses can be transported across the BBB by the cell–cell and accessory cell axons of the sensory and olfactory nerves.^80^ However, at present, the mechanism of the effect of COVID‐19 on the brain is not fully understood. Current studies found that COVID‐19 mainly induces CNS inflammation and neurodegenerative injury through a variety of pathological mechanisms (Figure 1).^81^

### 3.1 Direct infection injury

The neuronal retrograde pathway is one of the important ways for respiratory neurotropic viruses to invade the nervous system.^82^ In fact, peripheral nervous systems can provide a pathway for the virus to enter the nervous system.^83^ Based on this point, it is possible that SARS‐CoV‐2 could be transferred to the central nerve of the brain via the olfactory nerve. However, bioinformatics analysis of global and single‐cell RNA‐SEQ data sets of SARS‐CoV‐2 receptor expression in olfactory systems revealed that two key genes associated with SARS‐CoV‐2 invasion (ACE2 and TMPRSS2) were not expressed in olfactory neurons. This study suggested that SARS‐CoV‐2‐related proteins expressed in non‐neuronal olfactory system cells may lead to anosmia after COVID‐19 infection.^84^ Meanwhile, other peripheral nerves, such as neuromuscular junctions, may also contribute to neurological complications of COVID‐19. Significantly elevated levels of creatine kinase and myalgia or fatigue have been observed in many hospitalized COVID‐19 patients, further confirming that SARS‐CoV‐2 may invade myelin sheaths, or axons, of muscle neurons.^85^ In addition, the presence of COVID‐19 in CNS tissue samples, which indicated that the SARS‐CoV‐2 virus can also directly attack the CNS and lead to neurological damage. After entering the CNS, the SARS‐CoV‐2 virus interacts with the ACE2 of the neuron and enters neurons, initiating a period of virus germination in the cell and causing neuron damage at the same time. For example, many COVID‐19 patients present similar symptoms to intracranial infection (i.e., stroke and disturbance of consciousness), and some of these patients may develop infectious encephalitis, causing confusion.^34^

### 3.2 Hypoxia injury

When a person is infected with COVID‐19 and the virus proliferates in the patient's lungs, it results in diffuse alveolar damage and lymphocyte‐dominated inflammatory infiltration of the lung interstitium, which eventually leads to fibrous myxoid exudate edema and hyaluronic membrane formation,^29^ which disrupts the gas exchange in the alveoli, resulting in a lack of oxygen in the CNS. A hypoxic environment will increase the anaerobic metabolism of brain cells, resulting in an increase in acid metabolites, such as lactic acid, which will further cause cerebral vasodilation, brain cell swelling, interstitial edema, cerebral blood flow obstruction, and so forth. If hypoxia persists, it will further aggravate brain edema and cerebral circulation disorders, leading to headaches, drowsiness, bulbous conjunctival edema, and even coma and other symptoms of neurological injury.^81^

### 3.3 Immunologic injury

The virus interacts with immune cells to produce an immune response that is effective against virus invasion. It is well known that normal immune response can distinguish, recognize, and remove viruses as they enter the body, but an excessive immune response can lead to immune damage. Neurological damage by SARS‐CoV‐2 virus infection is closely related to the systemic inflammatory response syndrome (SIRS) caused by overactive immune responses. COVID‐19 has caused many patient deaths, which may be closely related to SIRS caused by immune overreaction or multiple organ failure caused by SIRS.^86^ In severe COVID‐19 patients, immune response results in SIRS, leading to neurological damage, so early anti‐inflammatory treatment in patients with immune overreaction may reduce immune injury and reduce neurological complications.^87^ The most common features of patients with severe COVID‐19 infection are lymphocytopenia and pneumonia, which are associated with congenital immune system dysfunction.^88^ Thus, abnormal immune system function in severe COVID‐19 patients may be due to a decrease in the number of CD4^+^ T cells. Meanwhile, as a reliable indicator of systemic inflammation and infection, an elevated neutrophil/lymphocyte ratio indicates the severe immune status in patients with severe COVID‐19. In addition, as SARS‐CoV‐2 is infected in the same way as SARS‐CoV and MERS‐CoV, serum levels of proinflammatory cytokines and chemokines are significantly elevated in blood samples from patients, suggesting that excessive inflammatory responses in severe COVID‐19 patients are justified.^89^ Interleukin‐6 (IL‐6), an important member of the cytokine storm, is positively correlated with the severity of COVID‐19 symptoms. IL‐6 secreted by macrophages causes neuroinflammation, and IL‐6 secreted macrophages are activated by granulocyte macrophage colony‐stimulating factor.^90^ The resulting cytokine storm leads to increased secretion of IL‐2, IL‐7, interferon‐γ monocyte chemotactic protein‐1, macrophage inflammatory protein‐1α, and tumor necrosis factor‐α, leading to an inflammatory response, which can lead to severe encephalopathy in patients.^91^ During the cytokine storm caused by COVID‐19 infection, some patients showed central neurological symptoms of ANE.^31^

There is increasing evidence that the innate immune system and inflammatory overreaction in patients with severe COVID‐19 are associated with respiratory failure ARDS and adverse clinical outcomes.^87^ The condition is thought to be a cytokine storm syndrome that contributes to vascular permeability, which can have a devastating effect on the pathological symptoms of COVID‐19 patients. The study found that cytokine storms cause disruption of the BBB, which protects the nervous system by controlling the entry of circulating molecules, immune cells, or viral particles into the system.^92^ In fact, the damaged BBB can offer a pathway for inflammatory mediators and immune cells to enter the brain. This aggressive invasion may present after SARS‐CoV‐2 infection, so brain inflammation may exacerbate the neurological symptoms associated with COVID‐19.^83^

## 4 CLINICAL TREATMENT OF NEUROLOGICAL SYMPTOMS IN COVID‐19 PATIENTS

Neurological symptoms can occur in many types of infections, and it may be difficult for medical staff to correlate the symptoms with COVID‐19 infection specificity. As a result, many neurological expressions in COVID‐19 patients have been overlooked, especially in the context of overburdened health resources. Hypogeusia and hyposmia are very important in the early stages of COVID‐19 disease, suggesting that these symptoms may involve the nervous system.^93^ In early cases of COVID‐19, changes in taste and smell were reported and no other complications were reported, indicating that the SARS‐CoV‐2 virus can transport into patients' nervous systems.^94^ However, the researchers found that patients without COVID‐19 infection also showed symptoms of anosmia and ageusia.

Generally, COVID‐19 patients experience respiratory symptoms before neurological symptoms. When COVID‐19 patients have shown neurological symptoms, it is important to do the test of COVID‐19 first before treating the patient for neurological disorders if it is necessary. In hospitals, medical workers should distinguish neurological emergencies from COVID‐19‐related emergencies, which can help prevent inadvertent exposure to emergency neurological COVID‐19 patients. When treating neurological patients without confirmed COVID‐19 disease, doctors should proactively ask patients if they have experienced COVID‐19 symptoms, such as fever and sore throat, in the past 2 weeks.^14^ Therefore, testing for COVID‐19 is very important, especially in the case of COVID‐19 infection.

Some COVID‐19 patients who are bedridden for long periods during mechanical ventilation may present myopathy or neuropathy following ARDS after hospitalization and may require extracorporeal membrane oxygenation therapy. In ARDS, the onset of debilitating, severe polymyopathy or polyneuropathy is often accompanied, which may require a multidisciplinary rehabilitation and recovery approach.^95^ For these reasons, early diagnosis and treatment of COVID‐19 patients are essential to prevent the virus from damaging the CNS of these patients.

## 5 LIMITATIONS OF THE LITERATURE ON COVID‐19 NEUROLOGICAL MANIFESTATIONS

With the quick spread of COVID‐19 around the globe, there is an urgent need for medical staff to be aware of the characteristics of COVID‐19 in the first place so that they can have early access to treatment experiences and methods for COVID‐19. As a result, the current study is filled with a large number of reports that perhaps falsely suggest a relation between COVID‐19 and CNS symptoms. In several research with very small sample sizes, about 1% of COVID‐19 patients have reported CNS complications, making it difficult to judge whether this is due to COVID‐19 or just a coincidence. In addition, aggressive use of anticoagulant therapy or treatment with extracorporeal membrane oxygenation may significantly increase the risk of cerebral hemorrhage, but this is not associated with COVID‐19.^96^ Currently, disturbance of consciousness is thought to be the result of COVID‐19 encephalopathy. It is worth pointing out that hypoxia due to lung injury in COVID‐19 patients also plays a role in neurological injury. Based on the above analysis results, only a large number of studies can effectively clarify the true pathogenic role of COVID‐19 in neurological complications. Therefore, researchers and medical staff should carefully consider the published results so far.

## 6 CONCLUSION

Up to now, COVID‐19 is still spreading worldwide, and people's understanding of COVID‐19‐related neurological diseases is further deepening. Neurological symptoms are a potential indicator of poor prognosis in COVID‐19 patients, and clinicians should continue to closely observe patients' neurological diseases. In addition, further studies on neuropathology are of vital importance for clinicians to have a deep understanding of the pathogenesis of COVID‐19 in the nervous system, which can provide a more sufficient theoretical basis for the subsequent clinical treatment of COVID‐19 and neurological diseases caused by it. This review summarizes and reviews the neurological diseases that can be caused by COVID‐19 infection, and further explores their possible pathogenesis, and provides clinicians with a series of recommendations for the management of COVID‐19 neurological complications. However, the understanding of the neuro erosive nature of the COVID‐19 virus is still very limited at present, and the research on its pathogenic mechanism is still the focus of current scientific research. Therefore, in the future, researchers will need to pursue multicenter, multifield collaborations to gain a deeper and more comprehensive understanding of COVID‐19's pathogenesis, which could guide the treatment of the disease.

## CONFLICT OF INTERESTS

The authors declare that there are no conflict of interests.

## ETHICS STATEMENT

Ethics statement is not applicable for the article.

## AUTHOR CONTRIBUTIONS

Mei‐Fang Xiao performed the data analyses and wrote the manuscript. Liang Dong and Ze‐Bing Huang contributed to the conception of the study. Chang Zeng contributed significantly to analysis and manuscript preparation. Zhi‐Jian You helped perform the analysis with constructive discussions.

## Data Availability

The data that support the findings of this study are available from the corresponding author upon reasonable request.
